# Radical Scavenging Activities of *Lagerstroemia speciosa* (L.) Pers. Petal Extracts and its hepato-protection in CCl_4_-intoxicated mice

**DOI:** 10.1186/s12906-016-1495-0

**Published:** 2017-01-18

**Authors:** Bipransh Kumar Tiwary, Somit Dutta, Priyankar Dey, Mossaraf Hossain, Anoop Kumar, Sony Bihani, Ashis Kumar Nanda, Tapas Kumar Chaudhuri, Ranadhir Chakraborty

**Affiliations:** 1 Department of Biotechnology, Omics Laboratory, University of North Bengal, Darjeeling, West Bengal 734013 India; 2 Department of Microbiology, North Bengal St. Xavier’s College, Rajganj, Jalpaiguri, 735135 India; 3 Department of Zoology, Cellular Immunology Laboratory, University of North Bengal, Darjeeling, West Bengal 734013 India; 4 Department of Chemistry, University of North Bengal, Darjeeling, West Bengal 734013 India; 5 ANMOL, Department of Biotechnology, University of North Bengal, Darjeeling, West Bengal 734013 India

**Keywords:** Antioxidant activity, *Lagerstroemia speciosa*, Hepatoprotective, CCl_4_-intoxicated, GC-MS

## Abstract

**Background:**

*Lagerstroemia speciosa* (L.) Pers. has medicinal importance. Bioactive phytochemicals isolated from different parts of *L. speciosa*, have revealed hypoglycemic, antibacterial, anti-inflammatory, antioxidant and hepato protective properties. Despite one report from Philippines detailing the use of *L. speciosa* as curative for fever and as well as diuretic, there is no experimental evidence about the hepatoprotective activity of the flower extracts.

**Methods:**

Several spectroscopic methods, including GC–MS, were used to characterize phytochemicals present in the petal extract of *L. speciosa*. Ethanol extract of petals was evaluated for anti-oxidant and free radical scavenging properties by using methods related to hydrogen atom transfer, single electron transfer, reducing power, and metal chelation. This study has also revealed the in vitro antioxidant and in vivo hepatoprotective properties of petal extract against carbon tetra chloride (CCl_4_)-induced liver toxicity in Swiss albino mice. Hepatoprotection in CCl_4_ -intoxicated mice was studied with the aid of histology and different enzymatic and non-enzymatic markers of liver damage. Cytotoxicity tests were done using murein spleenocytes and cancareous cell lines, MCF7 and HepG2.

**Result:**

GCMS of the extract has revealed the presence of several potential antioxidant compounds, of them γ-Sitosterol and 1,2,3-Benzenetriol (Pyrogallol) were the predominant ones. The antioxidants activities of the flower-extract were significantly higher than curcumin (in terms of Nitric oxide scavenging activity; *p* = 0.0028) or ascorbic acid (in terms of 2,2-Diphenyl-1-Picrylhydrazyl (DPPH) assay; *p* = 0.0022). The damage control by the flower extract can be attributed to the reduction in lipid peroxidation and restoration of catalase activity. In vitro cytotoxicity tests have shown that the flower extract did not affect growth and survivability of the cell lines. It left beyond doubt that a flower of *L. speciosa* is a reservoir of antioxidant and hepatoprotective agents capable of reversing the damage inflicted by CCl_4_-intoxication.

**Conclusion:**

Results from the present study may be used in developing a potential hepato-protective health drink enriched with antioxidants from *Lagerstroemia speciosa* (L.) Pers.

**Electronic supplementary material:**

The online version of this article (doi:10.1186/s12906-016-1495-0) contains supplementary material, which is available to authorized users.

## Background

*Lagerstroemia speciosa* (L.) is popularly called as “Jarul” in West Bengal, India and it belongs to the family Lythraceae. It is known as Pride of India, and also called Queen’s Flowers or Queen Crape Myrtle in English. This plant is widely distributed in the South East-Asian countries, Philippine and India [1]. In India, *L.speciosa* is highly abundant in the Western and Eastern Ghats and sub-tropical Himalayan regions; flowers are produced in excess by the plant (Additional file 1: Figure S1) for a short period of time but remains unutilized or underutilized. However, the people of South-east Asia used the leaves of *L. speciosa* for the treatment of diabetes mellitus and obesity [2]. The aqueous extract of leaves of *L. speciosa* leaves possess potent antioxidant and free radical scavenging activities by scavenging 2,2-Diphenyl-1-Picrylhydrazyl (DPPH) and superoxide radical as well as inhibiting lipid peroxidation [3]. Moreover, the bioactive phytochemicals isolated from different parts of *L. speciosa*, have revealed hypoglycemic, antibacterial, anti-inflammatory, antioxidant and hepato protective properties [4–9]. Flowers of several plants were reported as good source of phenolic compounds and antioxidants, and also reported for treating some chronic diseases reported by earlier authors [10]. In Philippines, the decoction of flowers of *L. speciosa* is used as diuretic and also for treating fevers [11, 12]. Hence, in this study we opted to explore the pharmacological properties of the flower extract of *L. speciosa*.

The mechanisms of generation of Reactive Oxygen Species (ROS), and scavenging of ROS, operate within living cells. However, damages are inflicted on several cellular macromolecules when there is an imbalance between the generation of ROS and the rate of scavenging. ROS have direct and indirect relationships with oxidation of cellular biomolecules resulting in many health disorders such as neurodegenerative disease, hypertension, inflammation, diabetes, cancer and aging [13]. Living organisms respond to ROS by producing antioxidant enzymes as well as they possess genetically regulated adaptive mechanisms against ROS. However, once the free radicals and ROS overwhelm the regulatory ability of the body, a state of oxidative stress ensues. Supplementation of anti-oxidants, in the normal diet, helps control the ROS-mediated macromolecular damages [14]. The use of natural compounds as complementary and alternative drug is on rise due to the lesser side effects compared to synthetic drugs. At present, natural antioxidants are also used as alternative to synthetic antioxidants in the cosmetic, pharmaceutical and in the food industries [15]. Moreover, presence of considerable quantity of antioxidants in Plant Part Extract (PPE) has always been a dependable clue for the investigators to hypothesize its usefulness in prevention and/or treatment of human diseases in which free radicals and other ROS have been associated. Therefore, hepatoprotective potentiality of PPE is generally evaluated against CCl_4_- induced liver damages in murine model [16, 17]. Several lead chemicals like silymarin, β-sitosterol, betalain, neoandrographolide, phyllanthin, andrographolide, curcumin, picroside, hypophyllanthin, kutkoside, and glycyrrhizin that have demonstrable hepatoprotective properties, were characterized from several PPEs [18]. High antioxidant activity in flower extracts of different plants such as *Tecoma stans*, *Hibiscus sabdariffa*, *Calendula officinalis*, and *Crocus sativus*, were screened for hepatoprotective activity by the previous research and proved viable. [19–22].

In the present study, in vitro antioxidant potential of 80% ethanolic extract of flower of *L. speciosa* was determined in addition to the quantification of phenolic and flavonoid contents. Prevention of hepatic cell damage by flower-extract in CCl_4_-intoxicated mice was demonstrated. Cytotoxicity tests of the flower-extract were conducted using murein spleenocytes and cancareous cell lines, MCF7 and HepG2. Since flower extract was found safe in cell-line study, we propose a future development of a suitable health drink from *L. speciosa* petals, a widely accessible natural bio-resource (Additional file 2: Figure S2).

## Methods

### Preparation of plant extract

The flowers were collected in the month of March (average number of flowers per tree remain higher than February or April) 2014, from *Lagerstroemia speciosa* (Jarul) trees within the campus of North Bengal University, West Bengal, India. The tree (Accession number- 10512) was authenticated by the Department of Botany, North Bengal University. The petals of the flower were separated and washed thrice with distilled water to remove dust. The washed petals were sun dried and treated at 50 °C for two hours to eliminate moisture. Dried petals were then milled with a grinder (Maharani, India, Model –Sujata Dynamix). The fine powdered petal was stored in a refrigerator at −20 °C. One hundred gm of the dried powder was stirred in 1 L of 80% ethanol for 1 hour. The mixture was refluxed for 2 hours in soxhlet. After 2 hours, the mixture was centrifuged at 8000 rpm for 15 minutes. Supernatant was collected and concentrated by Rotary evaporator (45 °C) and finally freeze dried. The extract was stored in air-tight vessel at −20 °C for further studies.

### Determination of antioxidant activity (in vitro)

#### In vitro assays

The total antioxidant, DPPH radical scavenging, hydroxyl radical scavenging, superoxide radical scavenging, nitric acid radical scavenging, singlet oxygen scavenging, reducing power, Fe^2+^ chelation, peroxynitrite scavenging and hypochlorous acid scavenging activities were determined by following the previous reported methods with minor modification[23, 24].

### Determination of erythrocyte-membrane stabilizing activity

The erythrocyte membrane stabilizing activity was performed by following a standard method as described by Dey et al. [25]. Briefly, varying concentrations of LFE (0–200 μg/ml) was added to the mixture of 50 mM phosphate buffer (0.5 ml; pH 7.2), distilled water (1 ml), 10% RBC suspension (0.25 ml PBS), 12 mM EDTA (100 μl), NBT (150 μl of 1% solution), and riboflavin (100 μl), and kept under bright light for 30 sec and incubated for 30 min at 50 °C followed by centrifugation at 1000 rpm for 10 min. The absorbance of the supernatant was measured at 562 nm. The same assay was done with the standard compound, quercetin.

### Determination of total phenolic content

The total phenolics content of LFE was determined using Folin-Ciocalteu method [23]. A standard curve prepared with known quantities of gallic acid (*R*_2_ = 0.9468) was used to measure the phenolic content of LFE.

### Determination of total flavonoid content

The total flavonoids content was determined with aluminium chloride (AlCl_3_) described by Hazra et al. [23]. The flavonoid content was ascertained from the standard curve prepared with known quantities of quercetin (*R*2 = 0.9947).

### Determination of cytotoxicity

#### MTT Cytotoxicity assay for murine spleenocytes

The spleen was separated from a sacrificed Swiss albino mice. Cell suspension (2 × 10^6^ cells/ml) was prepared in RPMI- 1640 medium supplemented with 50 U/ml penicillin, 50 U/ml streptomycin, 50 U/ml nystatin and 10% FBS as per reported method.EZcount ™ MTT Cell Assay Kit (HiMedia CCK003) was used, following manufacturers instruction, to determine the cytotoxicity. The percentage of cytotoxicity was calculated using the formula: (Y – X) ÷ Y × 100 [where Y is the mean optical density of the control (DMSO treated cells); and X is the mean optical density of the treated cells with LFE].

### Determination of effect of LFE on cancerous cells following MTT assay

The effect of LFE on cancerous cell lines was measured using a known MTT-assay protocol as described by Denizot & Lang [26] but with minor modifications. Two different cancerous cells, human breast adenocarcinoma cell line (MCF 7) and human hepatocarcinome cell line (HepG_2_) were obtained from National Centre for Cell Science, Pune, India. Both the cell lines were treated with different concentrations of LFE in this study.

### Determination of in-vivo antioxidant activity of LFE

#### Maintenance of Swiss albino mice

Swiss albino mice (6–8 weeks) of both sexes (equal number of mice from each sex) were maintained individually (one animal per cage in order to prevent aggression, if any, of one towards the other of the same sex or opposite) inside the cage bins (Tarson, India) with rice husk bedding in the animal enclosure of the Department of Biotechnology, University of North Bengal by maintaining proper photoperiod (12 h), temperature (25 ± 2^0^ C) and humidity (55 ± 5%). The animals were provided pellet food (Pranav Agro Pvt. Ltd. India) and filtered (Aquaguard Eureka Forbes) tap water *ad libitum*. All experiments were approved by the ethical committee University of North Bengal (NO.840/ac/04 CPCSEA; date: 15.09.2010).

### Determination of acute toxicity of LFE

Acute toxicity of LFE was studied following OECD in full guidelines (test 423: Acute oral toxicity – Acute toxic class method; 2002) [OECD Library]. Mice were divided into four groups (*n* = 6) and fasted overnight prior to the experiment. LFE was administered orally at 250, 500, 1000 and 1500 mg/kg body weight (bw) dose. The experimental mice were carefully observed for development of any clinical or toxicological symptoms at different time-period, 0.5, 2, 4, 8, 24 and 48 h.

### CCl_4_ intoxication of experimental mice followed by treatment with LFE or silymarin

Swiss albino mice, male or female, were randomly distributed into 5 groups (n = 6) and for consecutive 10 days they received treatments once per day as per design illustrated below.

The group that received normal saline was used as control. The other groups were : (i) CCl_4_ group which received 1:1 (v/v) CCl_4_ in olive oil; (ii) Silymarin group that received 1:1 (v/v) CCl_4_ in olive oil and 100 mg/kg bw silymarin; (iii) Lower dose (LD) of LFE treated group which received 1:1 (v/v) CCl_4_ in olive oil and 100 mg/kg bw LFE; and (iv) higher dose (HD) of LFE treated group which received 1:1 (v/v) CCl_4_ in olive oil and 250 mg/kg bw LFE.

After cardiac punctures of the anesthesized mice (for collection of blood) made on 11th day (i.e. 24 h after the last treatment), the animals were sacrificed. Blood was allowed to clot for 60 min at room temperature (20 °C) and then serum was separated by centrifuging at 1000 rpm for 5 min. Serum was used to study marker enzymes specific to liver. The liver was surgically removed from the anesthesized animals after the cardiac puncture and before the final sacrifice. Surgically separated livers were washed with double distilled water to remove blood and homogenized tissues were used for antioxidant enzymatic assays. Liver tissues were collected in Bouin’s solution for histological studies.

### Liver function test

The serum samples from each group were used to study Acid Phosphotase (ACP), Alkaline phosphatise (ALP), Aspartate aminotransferase (AST), Alanine aminotransferase (ALT) and total protein using commercially available kits (Biosystems; 11548, 11592, 11830, 11832, 11800).

### Determination of Catalase activity (CAT), lipid peroxidation activity (LPO) and reduced Glutathione (GSH) determination

CAT activity was measured by the method described by earlier authors [27]. Lipid peroxidation was quantified by thiobarbituric acid (TBA) reaction with malondialdehyde (MDA). The amount of MDA was assessed by measuring the absorbance of supernatant at 540 nm at room temperature against an appropriate blank [27]. Glutathione was determined by the modified method of Ellman [28].

### Histological studies

Livers were removed from the animals of the in vivo experiments after collection of blood and were fixed overnight in 10% buffered formalin. The samples were subjected to dehydration and the embedded in paraffin. Thin sections (4 μm) of the paraffin embedded livers were cut by microtome and then de-waxed in xylene, rehydrated in a series of different grades of alcohol and then washed with distilled water for 5 min. Subsequently, the sections were stained with haematoxylin for 40 s and counterstained with eosin for 20 s. The sections were dehydrated in graded alcohol series and washed in xylene. The slides were observed using Magnus trinocular microscope MLX-TR (Olympus microscopes) for signs of necrosis, portal inflammation, vascular congestion, fatty infiltration, vacuolar degeneration, leukocyte infiltration, loss of structure of hepatic nodules and so forth.

### Spectroscopic characterization of LFE

All UV–vis spectra were recorded in the range of 200–800 nm at room temperature with UV-1700 Spectrometer (Jasco Make, Tokyo, Japan). IR spectra of LFE obtained with Shimadzu FT-IR (Japan) were monitored by mulling in KBr. The Energy-dispersive Spectroscopy (EDS) was done with JEOL Model JED – 2300 to analyse the presence of different elements in the LFE.

### GC–MS analysis of LFE

LFE was dissolved in n-hexane and the mixture was centrifuged thrice at 12,000 rpm for 15 min. The clear supernatant was used for GC–MS analysis. Agilent 5975 CGCMS system (Agilent Technologies, USA) attached with HP-5 ms Capillary Column (30 m × 0.25 mm i.d. × 0.25 μm film thickness) and equipped with inert MSD triple axis mass detector condition edation trap 200 °C, transfer line 280 °C, electronenergy70eV (vacuum pressure-2.21e-0.5 Torr) was used for analysis. The carrier gas, helium, was used at a flow rate of 1 ml/min. 2 ml sample was injected in a split less mode. The column temperature was set at 60 °C for 1 min followed by 5 °C/min up to 250 °C. The major and essential compounds in LFE were identified by the retention times and mass fragmentation patterns using Agilent Chem Station integrator and the database of National Institute of Standard and Technology (NIST) with a MS library version2011.

### Statistical analysis

Assays were carried out in triplicate for all the experiments. The results are expressed as mean and standard deviation values (mean ± SD). Differences between means were determined by the analysis of variance (ANOVA), which were analyzed with SPSS v. 1. Paired ‘t’ test was done using Ky plot 5.0 (kyplot.software.informer.com/5.0/).

## Results

### In vitro antioxidant activity

**Fig. 1:**
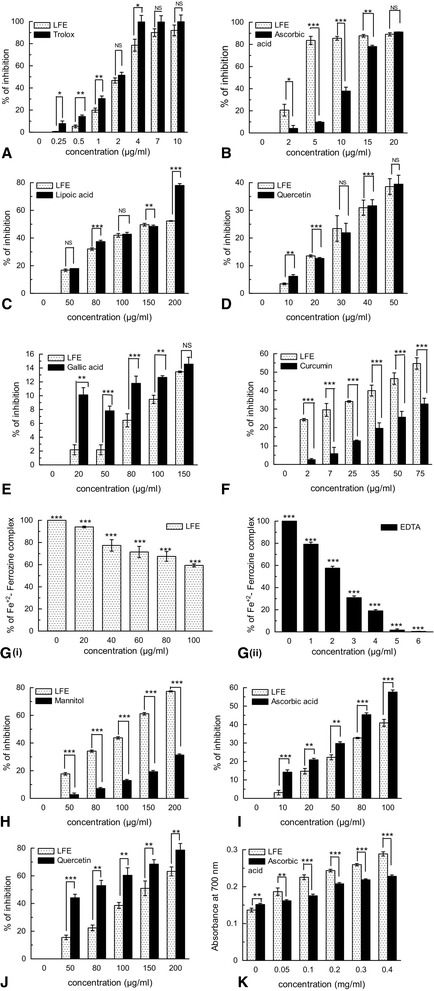
Free radical scavanging activity of *Lagerstroemia* flower extract (LFE). **a** Total antioxidant assay; **b** DPPH radical scavenging activity; **c** Singlet oxygen scavenging activity; **d** Superoxide radical scavenging activity; **e** Peroxynitrite radical scavenging activity; **f** Nitric oxide scavenging activity; **g** (i) and (ii). Fe chelation activity; **h** Hydroxy radical scavenging; **i** Hypocholorous radical scavenging activity; **j** Erythrocyte membrane stabilizing activity; and **k** Reducing power assay. Paired ‘t’ test was done to interpret significant difference between effect of LFE and the known standard; ***, *p* < 0.001; **, *p* < 0.01; and *, *p* < 0.05

The free radical scavenging activities of LFE in dose dependent manner and the differences in activities compared with standard compounds per test under varying doses were statistically interpreted (Fig 1a–k). The half maximal inhibitory concentration (IC_50_) of LFE or the corresponding reference compounds is shown in Table 1. LFE showed lower IC_50_ value than ascorbic acid, mannitol and curcumin in DPPH (*p* = 0.0022), hydroxyl radical (*p* = 0.00001) and nitric oxide free radical (*p* = 0.002) scavenging assays respectively; and found comparable with superoxide radical shown by quercetin (*p* = 0.52) and total antioxidant activity shown by trolox (*p* = 0.6).

**Table 1:** Half maximal inhibitory concentration (IC_50_) value of LFE and standards compounds for different free radical scavenging assays

S.No	Assay	Standard compound	Calculated IC_50_ Standard	Calculated IC_50_ LFE	2-sample t test (*p* value)
1	DPPH	Ascorbic acid	11.30 ± 1.23	3.23 ± 0.7	0.002
2	Superoxide radical	Quercetin	63.83 ± 2.5	65.57 ± 3.4	0.52
3	Singlet Oxygen	Lipoic acid	131.21 ± 8.3	162.72 ± 4.2	0.02
4	Total antioxidant assay	Trolox	3.26 ± 1.7	3.89 ± 0.5	0.6
5	Hypochlorous scavenging activity	Ascorbic acid	87.72 ± 4.9	124.03 ± 9.1	0.009
6	Hydroxyl radical	Mannitol	332.93 ± 3.5	124.75 ± 5.8	0.00001
7	Nitric oxide	Curcumin	109.60 ± 6.1	58.86 ± 7.5	0.0028
8	Peroxynitrite radical	Gallic acid	591.65 ± 13.9	500 ± 12.2	0.003
9	Erythrocyte membrane stabilizing activity	Quercetin	94.74 ± 5.5	152.48 ± 4.9	0.0008
10	Fe chelation	EDTA	25.37 ± 3.5	118.771 ± 12.4	0.0062

### Determination of reducing power

The reducing power of the LFE was determined. It was found that reducing capacity of the LFE was dose-dependent and comparable to the reference compound, ascorbic acid (Fig. 1k).

### Determination of Phenol content and flavonoids in LFE

The total amount of phenolic content present in ethanolic extracts of *L. specioa* was found to be 44.66 mg/ml gallic acid equivalent per 100 mg plant extract. The total flavonoid content of the LFE was 45.33 ± 0.004 mg/ml quercetin equivalent per 100 mg plant extract.

### Cytotoxicity and MTT assay

Treatment of cancerous cell lines, MCF-7 and HepG2, with LFE at different concentration from 0 to100 μg/ml showed no effect on the growth and survivability. Cytotoxicity of LFE was also evaluated by using murine spleenocytes and cytotoxic effect was not observed up-to treatment of 200 ug/ml of LFE in spleenocytes.

#### Hepatoprotective activity of LFE

##### Acute toxicity study

In the experimental mice, no signs of mortality were observed up to 1500 mg LFE/kg BW (highest dose used in this study). So, dosages of 100 mg/kg (low dose) and 250 mg/kg (high dose) were selected for the in-vivo hepatoprotective treatment.

#### Body and liver weight changes

Changes of the body and liver weight after the treatment of LFE are shown in Table 2. Significant weight loss was observed in CCl_4_ treated group whereas weight gain was observed in the control and silymarin group; but interestingly no significant weight gain was noticed in the experimental group. Hence, the percentage body weight change of CCl_4_ treated group was highest compared to the control, standard and experimental group.

**Table 2:** Comparision of body and liver weight of CCl_4_ induced with control (untreated), LFE treated and silymarin treated groups

Parameters (units)	Control	CCl_4_	Sylimarin	LFE Low Dose (100 mg/kg body weight)	LFE High Dose (250 mg/kg body weight)
Initial body weight (g)	21.70 ± 0.48	22.31 ± 0.26	22.37 ± 0.46	22.03 ± 0.46	22.07 ± 0.71
Final body weight (g)	23.28 ± 0.42	20.81 ± 0.74	23.31 ± 0.39	22.38 ± 0.70	22.17 ± 0.66
Body weight change (%)	6.79	6.74	4.20	1.59	0.45
Liver weight (g)	4.86 ± 0.11	5.46 ± 0.09	4.59 ± 0.22	5.03 ± 0.14	4.90 ± 0.08
Relative liver weight (g)	20.88	26.24	19.69	22.48	22.10

#### Liver marker enzyme and biochemical parameters

In this study, liver marker enzymes were estimated to obtain a clear picture of the medicinal potentiality of LFE in case of hepatic injury. The effects of CCl_4_ and subsequent administration of silymarin and LFE on the Acid phosphatase (ACP), Alkaline phosphatase (ALP), Aspartate transaminase (AST), Alanine transaminase (ALT) and protein level and percentage changes were shown in the Table 3. The levels of all the marker enzymes tested were found to be increased (except protein) on CCl_4_ administration and subsequently decreased with silymarin or LFE treatment.

**Table 3:** Extent of variation(s) in biochemical and enzymatic parameters in different groups treated with CCl_4_ or silymarin or LFE. The data represents mean ± SD of six independent observations

Parameters (Units)	Control	CCl_4_	Silymarin	LFE (Low Dose)	LFE (High Dose)
ALP (K.A.)	7.75 ± 0.17	28.52 ± 1.87 **	10.50 ± 1.61 ^NS^	18.48 ± 1.33 **	12.99 ± 0.22 **
ACP (K.A.)	4.06 ± 0.48	7.35 ± 0.11 **	16.55 ± 0.43 **	14.35 ± 0.34 ***	10.79 ± 0.50 **
AST (u/ml)	61.78 ± 2.48	133.21 ± 3.94***	75.26 ± 3.29 ***	126.27 ± 2.73 ***	86.12 ± 4.29 ***
ALT (u/ml)	51.27 ± 1.58	138.29 ± 4.83 ***	68.28 ± 3.97 ***	109.93 ± 3.75 ***	81.28 ± 4.17 ***
Protein (g/dl)	7.61 ± 0.27	4.42 ± 0.30 *	7.40 ± 0.30 ^NS^	4.74 ± 0.10 **	5.50 ± 0.19 *

N.S. *P* > 0.05 When compared with control, *P < =0.05 When compared with control, ** *P* < =0.01 When compared with control, *** *P* < =0.001 When compared with control

#### Lipid peroxidation (LPO), enzymatic catalase (CAT), and non-enzymatic reduced glutathione (GSH) level antioxidant assays

**Fig. 2:**
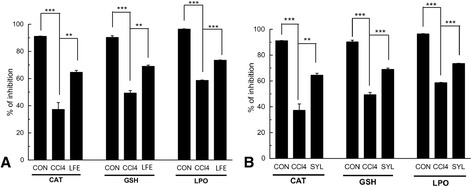
Hepatoprotective effect of *Lagerstroemia* flower extract (LFE) or silymarin (SYL) in CCl_4_ treated mice. **a** Protective effect of LFE on catalase (CAT) activity, reduced glutathione (GSH) and Lipid peroxidation (LPO) in CCl_4_ treated mice. **b** Protective effect of silymarin on catalase (CAT) activity, reduced glutathione (GSH) and Lipid peroxidation (LPO) in CCl_4_ treated mice group. Comparisons were made with (i) control (CON); (ii) CCl_4_ treated (no protection) (CON) for statistical inference (‘t’ test for paired comparison) to interpret significant difference (Data represented as Mean ± SD of six observations. *, *p* < 0.05, **, *p* < 0.01 and ***, *p* < 0.001.)

Significant inhibitions of LPO (*p* ≤ 0.001), enzymatic CAT (*p* ≤ 0.005) and non – enzymatic GSH (*p* ≤ 0.001) occurred in CCl_4_ intoxicated mice when compared with control (Fig. 2). LFE treatment enabled significant increase in % inhibition of LPO (*p* ≤ 0.001), CAT (*p* ≤ 0.01) and GSH (*p* ≤ 0.01) compared to CCl4 treated mice (Fig. 2a).On the other hand, silymarin treatment has similarly led to significant increase in % inhibition of LPO (*p* ≤ 0.001), CAT (*p* ≤ 0.01) and GSH (*p* ≤ 0.001) compared to CCl4 treated mice (Fig. 2b).

#### Histological comparison between liver tissue of CCl_4_-intoxicated and CCl_4_-intoxicated but silymarin or LFE treated mice

**Fig. 3:**
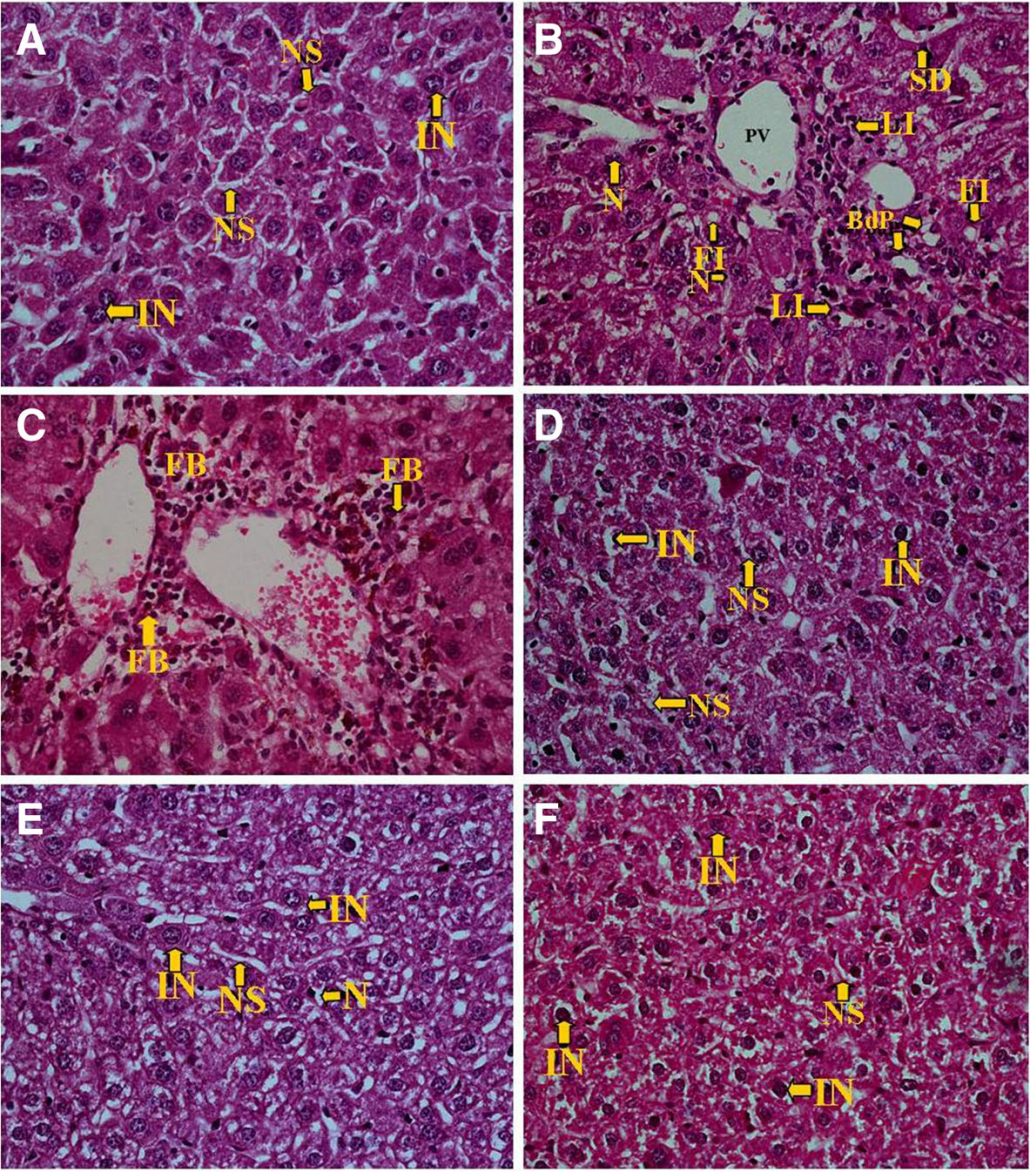
Photomicrographs: histological sections of mice liver samples. Pictures were taken under original magnification of 400X. **a** Liver section from the control group demonstrating normal liver architecture with intact nucleus (IN), and normal sinusoids (NS); **b** Liver section from CCl_4_ induced damaged liver demonstrating highly deformed liver architecture with round congested portal vein (PV), bile duct proliferation (BdP), fatty lesion due to intensive fatty infiltration (FI), sign of necrosis (N), dilated sinusoid (SD), leukocyte infiltration (LI); **c** Liver section from CCl_4_ induced damaged liver demonstrating fibrosis (FB); **d** Liver section from Silymerin treated group demonstrating improved hepato-cellular architecture with normal sinusoids and intact nucleus (IN); **e** Liver section from low dose LFE (100 mg/kg of body weight) treated group showing sign of necrosis (N) [of lesser degree compared to the CCl_4_ group]; **f** Liver section from high dose LFE (250 mg/kg of body weight) treated group showing improved liver architecture with normal sinusoids (NS) and intact nucleus (IN)

The histological injury was observed and counts in the liver tissue of CCl_4_-intoxicated and CCl_4_ -intoxicated but silymarin or LFE treated (low dose or high dose) mice were represented as injury score (Additional file 3: Table S1). The haematoxalin – eosin staining of liver tissue sections clearly displayed differences resulting from damages inflicted by CCl_4_. The liver tissue sections of the control group showed well maintained hepatocellular integrity, healthy cellular architecture, and clear cytoplasm with prominent nucleus (Fig 3a) while signs of tissue damages were evident in CCl_4_ treated mice liver sections (Fig. 3b) including signs of fibrosis (Fig. 3c and Additional file 4: Figure S4). On treatment of silymarin, the signs of healing of the damaged tissue were evident (Fig. 3d). Low dose treatment (100 mg/kg body weight) of LFE helped to reduce the damage but to a lesser extent when compared to silymarin treatment (Fig. 3e). Comparatively, treatment with higher dose of LFE (250 mg/kg body weight) has shown better recovery (Fig. 3 f). Total damage score was very high in CCl_4_ intoxicated mice (23) compared to control (2), silymarin group (7) and LFE treated group LD (16) and HD (9). (Additional file 3: Table S1).

#### Furier Transform Infrared (FTIR) spectroscopy

On analyses of FTIR spectra several intense peaks corresponding to the defined functional groups were noted. It indicated the presence of alcohols, phenols, carboxylic acid, within range of 3000–3550 cm^−1^, aldehydes, ketones, carboxylic acid at 1708 cm^−1^, amide bonds at 1604 cm^−1^, amines, sulfones, sulfomyl chloride at 1316 cm^−1^, alcohols, and carboxylic acids at 1176 cm^−1^ (Additional file 5: Figure S5).

#### Energy dispersion spectroscopy

The elemental composition of the LFE was determined by EDS (Additional file 6: Figure S6). The intense signals in the range of 0–0.5 keV –strongly suggests that carbon and oxygen were the major elements.Additionally, peak for potassium element was also found. Again the ED spectra have revealed absence of heavy metal.

#### GC–MS analysis

GC–MS analysis (Additional file 7: Figure S7) of LFE has enabled identifying several small compounds of diverse chemical nature (Table 4), of which many of them are reported to possess distinct and definitive pharmacological activities.

**Table 4:** Phytochemicals identified in the ethanolic extract of the flower of *L.speciosa* by GC-MS analysis

Sl. No.	Compound name	Chemical formula	RT
1.	2,6-Nonadienal, 3,7-dimethyl-	C_11_H_18_O	5.60
2.	N-[4-(4-Chlorophenyl)isothiazol-5-yl)-1-methylpiperidin-2-imine	C_15_H_16_ClN_3_S	5.84
3.	2-Furancarboxaldehyde, 5-methyl-	C_6_H_6_O_2_	6.77
4.	Formamide, N-[1-[(1-cyano-2-methylpropyl) hydroxyamino]-2-methylpropyl]-	C_10_H_19_N_3_O_2_	6.94
5.	Oxazolidine, 2,2-diethyl-3-methyl-	C_8_H_17_NO	6.74
6.	Oxirane, [(hexadecyloxy)methyl]-	C_19_H_38_O_2_	9.27
7.	2H-Tetrazole, 2-(1,3-dioxolan-4-ylmethyl)-	C_6_H_10_N_4_O_2_	9.76
8.	Furylhydroxymethyl ketone	C_6_H_6_O_3_	9.94
9.	2,3-Dimethylfumaric acid	C_6_H_8_O_4_	10.46
10.	4H-Pyran-4-one, 2,3-dihydro-3,5-dihydroxy-6-methyl-	C_6_H_8_O_4_	11.63
11.	2-Furanone, 3,4-dihydroxytetrahydr	C_4_H_6_O	12.96
12.	d-Ribo-hexos-3-ulose	C_6_H_10_O_6_	13.62
13.	5-Hydroxymethylfurfural	C_6_H_6_O_3_	13.97
14.	d-Mannose	C_6_H_12_O_6_	14.89
15.	Tetradecanoic acid, 2-hydroxy-	C_14_H_28_O_3_	15.25
16.	5-(Hydroxymethyl)-2-(dimethoxymethyl) furan	C_8_H_12_O_4_	15.87
17.	1,2,3-Benzenetriol (Pyrogallol)	C_6_H_6_O_3_	17.93
18.	Desulphosinigrin	C_10_H_17_NO_6_S	19.49
19.	D-Allose	C_6_H_12_O_6_	20.64
20.	3-tert-Butyl-4-hydroxyanisole (also known as 3-BHA, which is a potent antioxidant)	C_11_H_16_O_2_	22.66
21.	Benzoic acid, 4-hydroxy-3,5-dimethoxy- (also known as Syringic acid)	C_9_H_10_O_5_	28.09
22.	n-Hexadecanoic acid	C_16_H_32_O_2_	31.03
23.	Hexadecanoic acid, ethyl ester	C_18_H_36_O_2_	31.70
24.	9,12-Octadecadienoic acid (Z, Z)- (also known as Linoleic acid)	C_18_H_32_O_2_	34.23
25.	9,12,15-Octadecatrienoic acid, 2,3-dihydroxypropyl ester, (Z, Z, Z)-	C_21_H_36_O_4_	34.34
26.	γ-Sitosterol	C_29_H_50_O	52.51

## Discussion

Interest in antioxidants of natural origin as food and health supplements has increased much because of their potential to prevent and to reduce the risk of several diseases without any toxic effect [29]. The plant species, *L. speciosa* (L.) Pers, in the 1990’s, has attracted attention of the scientists worldwide because of its special therapeutic properties particularly for diabetes, obesity, and renal disorders [30, 31]. Although, different vegetative parts as well as seeds of this plant were explored for potential antioxidant agents [6, 7] but, only a single report exists that has mentioned the antioxidant activity of *L. speciosa* flowers [32]. Generally, antioxidant activities present in the plant extracts are studied with reference to hydrogen atom transfer (HAT), single electron transfer (ET), reducing power, and metal chelation assays [33]. Therefore, in the screening of antioxidant activity of LFE, it showed strong scavenging capacity against DPPH radical, singlet oxygen, superoxide radical, NO- radicals and hydroxyl radical in a dose dependent way (Fig. 1 and Table 1). Total antioxidant activity of LFE’s was found similar to trolox (standard compound) in neutralizing the radical cation ABTS^•+^ (Fig. 1a). Hypochlorous acid is known to get produced from the site of inflammation resulting from the oxidation of Cl^−^ ions by the neutrophil enzyme, myelo-peroxidase. The radical, HOCl is known to degrade heme-prosthetic group and inactivate the antioxidant enzyme, catalase. The HOCl scavenging activity of the LFE corresponded with the inhibition of catalase deactivation (Fig 1i and Table 1).

Reducing power is also one of the measures to confirm antioxidant activity and thus could serve as an indicator of potential antioxidant activity [34]. In this study, the reducing power of LFE was found comparable with standard compound ascorbic acid (Fig. 1k). It was conjectured that compounds with chelating activity can inhibit lipid peroxidation by stabilizing transition metals. Our results have indicated that the chelating effect of LFE would be at least partly beneficial in protecting against oxidative damage, but not efficient as EDTA. The results also showed that LFE could protect erythrocyte membrane stabilizing activity better than the standard compound quercetin by means of scavenging superoxide radicals (Table 1).

Our results revealed the presence of high contents of phenolic and flavonoids in LFE, which is similar to an earlier report [32]. Phenolics and flavanoid compounds are capable of scavenging singlet oxygen and various free radicals [35]. They may also help to prevent diseases associated with oxidative stress, such as atherosclerosis, cancer and neurodegenerative diseases [36]. In this study, results of cytotoxic activity in murine spleenocytes and human MCF 7 and HepG2 cell lines have shown no inhibition in growth, thus ruling out toxic effect of LFE on mammalian cells (data not shown). Taken together all the results, we may say with caution that LFE is perhaps safe for human consumption.

Moreover, it is essential to confirm in vitro results with in vivo assays. A common hepatotoxin, CCl_4_, is generally used to induce hepatic damage in animal model to understand the extent of tissue damages for correlating conditions that happen in human beings during acute hepatitis [37]. In this perspective, we have used mice as a model animal to check CCl_4_-induced hepatoxicity and subsequent hepato-protection with the aid of LFE. When mice is fed with CCl_4_, cytochrome P450 (liver enzyme) metabolises it to two trichloromethyl radicals, CCl^.^_3_ and CCl3OO^.^, by cleaving the carbon chloride bond of carbon tetrachloride [38]. The trichloromethyl radicals generated from CCl_4_ initiate free radical-mediated lipid peroxidation, which in turn leads to the accumulation of oxidation products causing apoptosis or necrosis in liver tissues [39]. We have found that LFE can heal CCl_4_ induced damaged liver in mice (Fig. 3). In case of acute hepatic damage (due to toxicity) in human beings, silymarin, an antioxidant flavanoid, is prescribed as a healing agent [40, 41]. The same compound, sylmarin, was used as the control preventive agent in our experiment. Results have shown that exposure to CCl_4_ caused significant difference in body, liver and relative liver weights with respect to the control group. Reduction in body weight and increment in liver weight took place in CCl_4_ intoxicated mice with respect to the control group. Due to CCl_4_ toxicity, relative liver weight of CCl_4_ treated mice was found much higher than the control (Table 2). It is known that liver weight generally increases due to hepatic damage inflicted by trichloromethyl radical [42]. Liver weight may also increase due to consequent liver fibrosis; and hypertrophy could therefore arise due to accumulation of glycogen in hepatocytes [43]. Hence, changes in body and liver weight after CCl_4_ intoxication provides direct evidence to the overall hepatic damage. Treatment with LFE (250 mg/kg body weight) has significantly prevented subsequent liver enlargement in mice. Lowering of liver or relative liver weight in LFE treated mice compared to CCl_4_ group reflected prevention of fatty liver formation on CCl_4_ toxicity. On other hand, weight gain was restricted in LFE treated group as compared to control (untreated) groups (Table 2), for which no definite explanation could be made; and it may be due to presence of some anti-diabetic and anti obesity compounds in LFE.

It is known that in case of extensive hepatic damages, enzymes, like AST and ALT, leave the confinement (within liver tissue) and escape into the circulatory system [44, 45]. Hence, we have studied the levels of AST and ALT in the serum of the diseased mice compared to the untreated control. Serum AST and ALT levels were found to increase markedly in CCl_4_ intoxicated mice clearly indicating altered permeability of membranes and hepatotoxicity. Interestingly, the level of AST and ALT were significantly reduced by administration of LFE (Table 3). Thus it was revealed that LFE can increase the structural integrity/stabilization of plasma membrane, which also supported the in-vitro erythrocyte membrane stabilizing activity. Moreover, restoration of structural cell integrity in case of treatment with LFE was supported by histology (by comparing the histological sections, Fig. 3). To understand more about the hepatoprotective effect rendered by LFE, the total protein concentration was measured. Total protein level, which came down, in CCl_4_ intoxicated mice was partially restored by treatment with LFE. The role of antioxidant activities of LFE in vivo was studied by measuring activities of antioxidant enzymes catalase (CAT) and the levels of GSH and TBARS in the liver. TBARS (markers of lipid peroxidation) is used as a main marker of hepatocellular injury [46]. Moreover, peroxidation of polyunsaturated fatty acids at the cell membrane leads to a cytotoxic by-product, malondialdehyde (MDA). During oxidative stress in liver, the amount of MDA determines the extent of oxidative damage [47]. A lower MDA value in liver tissue of mice indicated a stronger protective activity in samples. Our results have shown higher concentration of MDA in CCl_4_ treated group while silymerin or LFE (High dose) group significantly reversed these changes through reduction of lipid peroxidation and decreased production of free radical derivatives. This inference was substantiated by the observed decreased level of TBARS. GSH (non-enzymatic antioxidants) is the major non-protein thiol that plays a vital role in maintaining the body’s antioxidant defence mechanism [48, 49]. It was found that the level of GSH in the liver dropped down in CCl_4_ intoxicated mice. It is of general perception that accessibility of the liver cells to potential antioxidant molecules may prevent gross depletion of GSH to save the organ from destruction by free radical assault. In our case, perhaps, feeding of LFE has probably played an important role in restoring the normal intracellular GSH level. Catalase is an antioxidant enzyme which promotes the degradation of H_2_O_2_ into water and oxygen [50]. Inhibition of enzymatic activities like catalase activity cause accumulation of superoxide radical and H_2_O_2_, which attenuates a cascade of free radical formation. Catalase was found to be increased in LFE (High Dose) or silymerin treated group compared to CCl_4_ treated group (Fig 2 a and b). This restoration of catalase activity in LFE indicated the potential of LFE as antioxidant and was thus comparable to the known antioxidant, silymerin. These findings have clearly indicated that LFE is capable of protecting the liver by means of improving the enzymatic and non-enzymatic antioxidant defense systems, thus significantly reducing the generation of in vivo free radicals activated by CCl_4_. Histopathological observations have provided phenotypic support in favour of LFE’s hepato-protective role in curbing the intensity of damage done by CCl_4_ intoxication. The occurrence of various signs of liver injury (Additional file 3: Table S1) confirmed extensive hepatic tissue damage in CCl_4_ group. CCl_4_ intoxication led to tissue degeneration in liver, which was clear from prominent signs of necrosis. Silymarin and LFE administration demonstrated regeneration of healthy liver tissue with much lesser signs of injury as compared to CCl_4_ treated group. The microscopy has enabled to distinguish between prominent nucleus containing organized hepatocytes (control) and the deformed nucleus in ameboid overlapped hepatocytes observed in CCl_4_ treated mice’s liver (Fig 3). Restoration of tissue integrity (tight packed cells) was also observed in Silymarin or LFE group. The fatty infiltrations, due to lipid peroxidation, were prominent in CCl_4_ group, but found lower in silymerin or LFE (High Dose) treated ones. Nevertheless, treatment with LFE demonstrated prominent restoration in hepatocytes. The reduced cytoplasm vacuolization, mononuclear infiltration, prevention of necrosis, and normalized sinusoidal spaces established the hepatoprotective potential of LFE in recovering normal hepatic histoarchitecture.

The antioxidant components present in the LFE was correlated with GCMS data (Additional file 5: Figure S5). The phytochemicals, sitosterol, 1,2,3-benzenetriol (pyrogallol), 3-tert-butyl-4-hydroxyanisole (also known as 3-BHA), syringic acid, oxazolidine-2, 4-dione, 9,12- Octadecadienoic acid and furan-2- carboxylic acid-3-methyl- trimethyl silyl ester identified from the GCMS data (Table 4) have reported antioxidant activities [51–56]. Sitosterol has anti-hepatotoxic activities which normalizes serum transminase and hepatic antioxidant enzymes in hepato-compromised animals [55]. There may be some more phytochemicals (remained in the GCMS data beyond the known ones) in LFE which are yet to be identified as hepatoprotective agents. In one of our previous reports, it was shown that multiple constituents of a PPE may act synergistically or additively to affect the biological system [57]. There are also other reports on using combination of compounds to gain higher therapeutic effectiveness over singly administered compound(s) [58]. Based on this philosophy, we propose the therapeutic prospect of the flower extract of *Lagerstroemia speciosa* (L.) Pers in treating liver damages (Additional file 2: Figure S2).

## Conclusion

This study has revealed the antioxidant activity of *L. speciosa* flower extract (LFE), more comprehensibly, by conducting in-vivo studies in addition to in vitro tests. LFE can scavenge or neutralize free radicals of different origin and chelate ferrous ion. There was no toxic effect of LFE on murine spleenocytes and human MCF7 and HepG2 cell lines. The in-vivo tests have indicated that feeding of LFE has several manifestations, like reduction of MDA level, increase in GSH level, and restoration of catalase in CCl_4_ intoxicated mice, to reverse liver damage to a considerable extent. Furthermore, GCMS analyses have confirmed the presence of various compounds reported as potential antioxidant. These compounds may have contributed towards protection against damages inflicted by free radicals. Summing up all the properties shown by LFE, *L. speciosa* flowers could be a promising candidate as functional food, obviously after satisfying FDA recommendations. The fact remains that even in the face of rapid urbanization, majority of Indians live in the villages. There are several tribal pockets. Liver damages (both alcoholic and non-alcoholic damaged liver patients are innumerable) are rampant among the rural people. Popularizing such drink which people themselves can prepare will be beneficial to the society at large (Additional file 2: Figure S2).

## Additional files

Additional file 1: Figure S1. Tree view and flowers of *Lagerstroemia speciosa* (a) *L. speciosa* in full bloom in the month of March 2014 (b) Flowers of *L. speciosa* (*DOCX 289 kb*)
Additional file 2: Figure S2. Graphical Abstract of the prospective development of a health drink from flower extract of *Lagerstroemia speciosa* (*DOCX 522 kb*)
Additional file 3: Table S1. Comparative scoring of liver histology parameters of the CCl_4_ induced mice (injured liver) with control (untreated) and treated (LFE). (DOCX 11 kb)
Additional file 4: Table S4 Photomicrographs of the histological examination of livers samples. (A) Control group demonstrated, normal liver architecture (magnification of 100X); (B) CCl_4_ group liver demonstrated fibrosis (FB) (magnification of 100X) (C) and (D) CCl_4_ group liver demonstrated fibrosis (FB) (magnification of 400X) (DOCX 178 kb)
Additional file 5: Figure S5: FTIR spectra of flower extract of *Lagerstroemia speciosa* (*DOCX 44 kb*)
Additional file 6: Figure S6. EDS spectra of ethanolic flower extract of *Lagerstroemia speciosa* (*DOCX 58 kb*)
Additional file 7: Figure S7. GCMS spectra of lyophilysed ethanolic flower extract of *Lagerstroemia specios* (*DOCX 125 kb*)
