# Supplementary material for: Radical Scavenging Activities of Lagerstroemia speciosa (L.) Pers. Petal Extracts and its hepato-protection in CCl4-intoxicated mice
Source: BMC Complement Altern Med. 2017 Jan 18;17:55. doi: 10.1186/s12906-016-1495-0 (PMC5241977; doi:10.1186/s12906-016-1495-0)

**Additional file 1**

**Tree view and flowers of *Lagesteromia speciosa*** (a) *L. speciosa* in full bloom (b) Flowers of *L. speciosa*

(a)


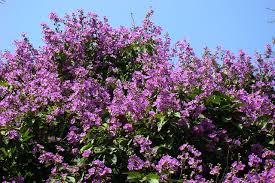


(b)


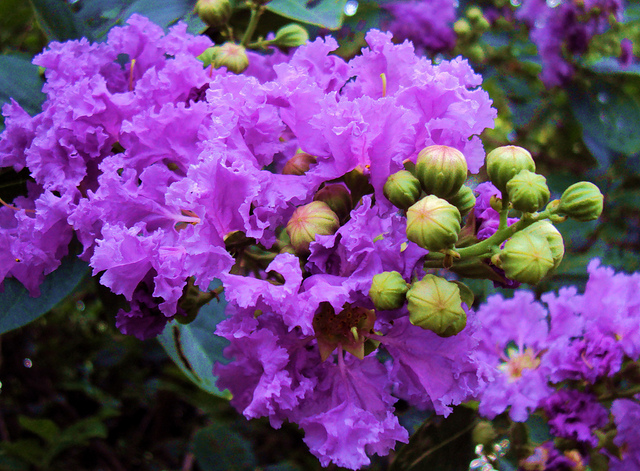

Supplement: Additional file 1: Figure S1. — Tree view and flowers of Lagerstroemia speciosa (a) L. speciosa in full bloom in the month of March 2014 (b) Flowers of L. speciosa (DOCX 289 kb) [file 12906_2016_1495_MOESM1_ESM.docx]
