# Supplementary material for: Radical Scavenging Activities of Lagerstroemia speciosa (L.) Pers. Petal Extracts and its hepato-protection in CCl4-intoxicated mice
Source: BMC Complement Altern Med. 2017 Jan 18;17:55. doi: 10.1186/s12906-016-1495-0 (PMC5241977; doi:10.1186/s12906-016-1495-0)

**Additional file 2**

Graphical Abstract of the prospective development of a health drink from petal extract of *L. Speciosa*


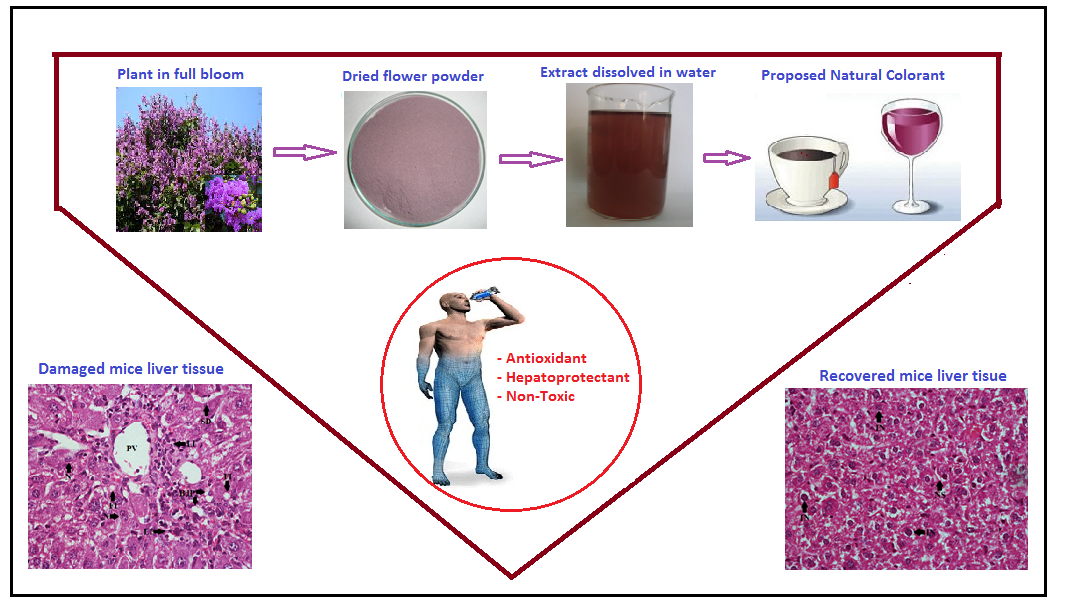

Supplement: Additional file 2: Figure S2. — Graphical Abstract of the prospective development of a health drink from flower extract of Lagerstroemia speciosa (DOCX 522 kb) [file 12906_2016_1495_MOESM2_ESM.docx]
