# Supplementary material for: Radical Scavenging Activities of Lagerstroemia speciosa (L.) Pers. Petal Extracts and its hepato-protection in CCl4-intoxicated mice
Source: BMC Complement Altern Med. 2017 Jan 18;17:55. doi: 10.1186/s12906-016-1495-0 (PMC5241977; doi:10.1186/s12906-016-1495-0)
Supplement: Additional file 3: Table S1. — Comparative scoring of liver histology parameters of the CCl4 induced mice (injured liver) with control (untreated) and treated (LFE). (DOCX 11 kb) [file 12906_2016_1495_MOESM3_ESM.docx]

**Additional File 3**

**Comparative scoring of liver histology parameters of the CCl_4_ induced mice (injured liver) with control (untreated) and treated (LFE).**

| **Parameters studied** | **Control** | **CCl_4_** | **Silymarin** | **LFE low** | **LFE High** |
| --- | --- | --- | --- | --- | --- |
| Hepatocellular necrosis | 0 | 6 | 2 | 4 | 2 |
| Bile adduct prolifreration | 0 | 1 | 0 | 2 | 0 |
| Sinusoidal dilatation | 0 | 1 | 0 | 1 | 0 |
| Inflammation (leukocytes infiltration) | 2 | 8 | 4 | 6 | 5 |
| Loss of structure of hepatic nodules | 0 | 3 | 1 | 2 | 2 |
| Heapatocellular fibrosis | 0 | 1 | 0 | 0 | 0 |
| Fatty infiltration | 0 | 1 | 0 | 0 | 0 |
| Calcification | 0 | 2 | 0 | 1 | 0 |
| Cumulative score | 2 | 23 | 7 | 16 | 9 |
