# Supplementary material for: Radical Scavenging Activities of Lagerstroemia speciosa (L.) Pers. Petal Extracts and its hepato-protection in CCl4-intoxicated mice
Source: BMC Complement Altern Med. 2017 Jan 18;17:55. doi: 10.1186/s12906-016-1495-0 (PMC5241977; doi:10.1186/s12906-016-1495-0)

**Additional file 4**

Photomicrographs of the histological examination of livers samples. (A) Control group demonstrated, normal liver architecture (magnification of 100X); (B) CCl_4_ group liver demonstrated fibrosis (FB) (magnification of 100X ) (C) and (D) CCl_4_ group liver demonstrated fibrosis (FB) (magnification of 400X ).


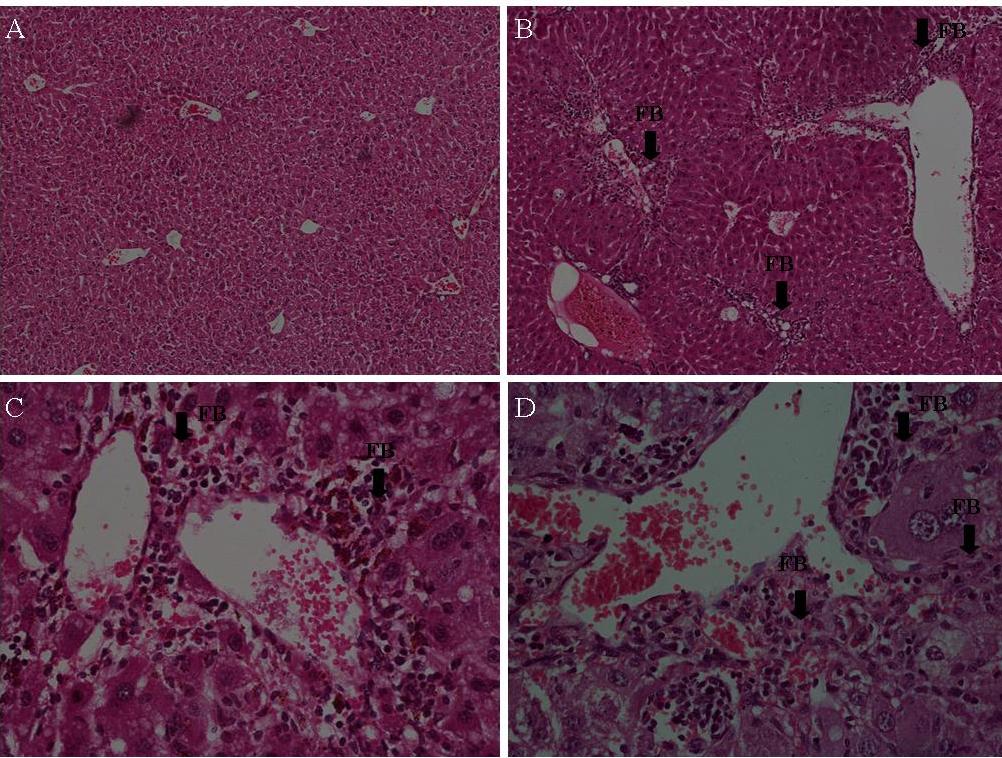

Supplement: Additional file 4: Table S4 — Photomicrographs of the histological examination of livers samples. (A) Control group demonstrated, normal liver architecture (magnification of 100X); (B) CCl4 group liver demonstrated fibrosis (FB) (magnification of 100X) (C) and (D) CCl4 group liver demonstrated fibrosis (FB) (magnification of 400X) (DOCX 178 kb) [file 12906_2016_1495_MOESM4_ESM.docx]
