# Supplementary figures and images for: Radical Scavenging Activities of Lagerstroemia speciosa (L.) Pers. Petal Extracts and its hepato-protection in CCl4-intoxicated mice
Source: BMC Complement Altern Med. 2017 Jan 18;17:55. doi: 10.1186/s12906-016-1495-0 (PMC5241977; doi:10.1186/s12906-016-1495-0)

**Additional file 5**

FTIR spectra of flower extract of *Lagerstroemia speciosa*


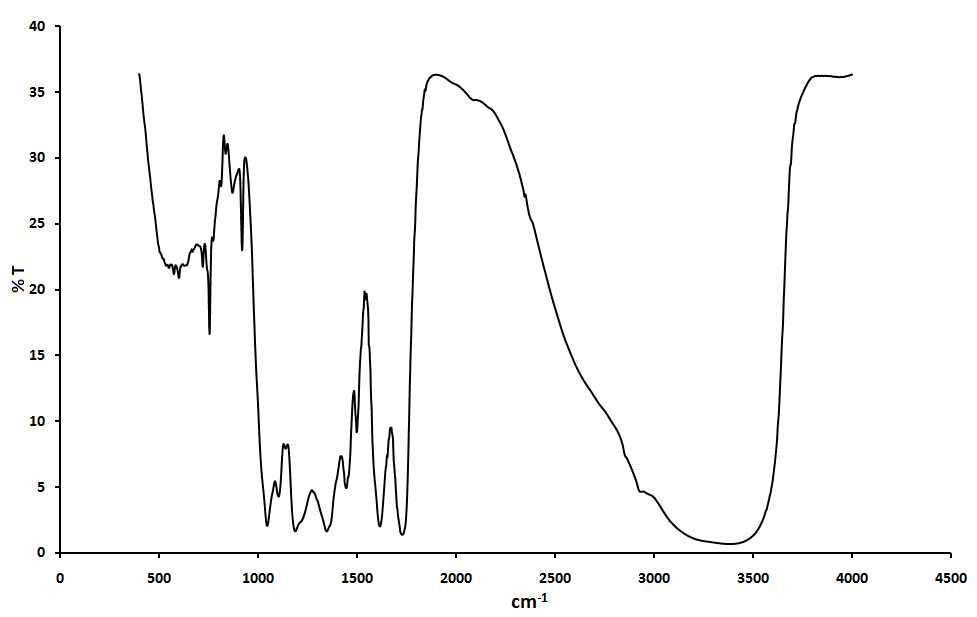

Supplement: Additional file 5: Figure S5: — FTIR spectra of flower extract of Lagerstroemia speciosa (DOCX 44 kb) [file 12906_2016_1495_MOESM5_ESM.docx]

**Additional file 6**

EDS spetra of flower extract of *Lagerstroemia speciosa*


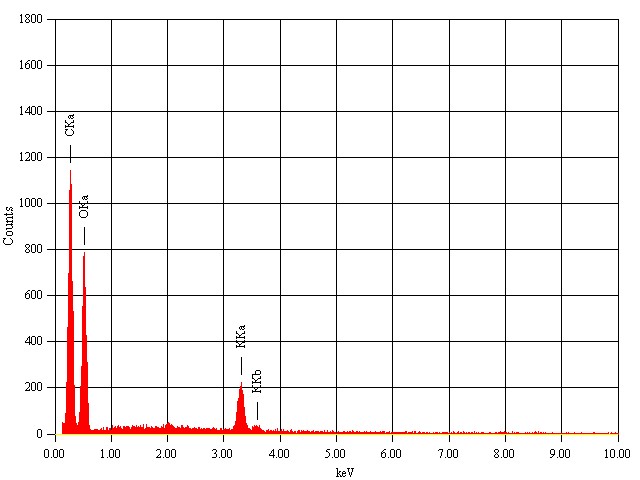

Supplement: Additional file 6: Figure S6. — EDS spectra of ethanolic flower extract of Lagerstroemia speciosa (DOCX 58 kb) [file 12906_2016_1495_MOESM6_ESM.docx]
