# Supplementary material for: Radical Scavenging Activities of Lagerstroemia speciosa (L.) Pers. Petal Extracts and its hepato-protection in CCl4-intoxicated mice
Source: BMC Complement Altern Med. 2017 Jan 18;17:55. doi: 10.1186/s12906-016-1495-0 (PMC5241977; doi:10.1186/s12906-016-1495-0)

**Additional file 7**

GCMS spectra of lyophilysed ethanolic flower extract of *Lagerstroemia speciosa*


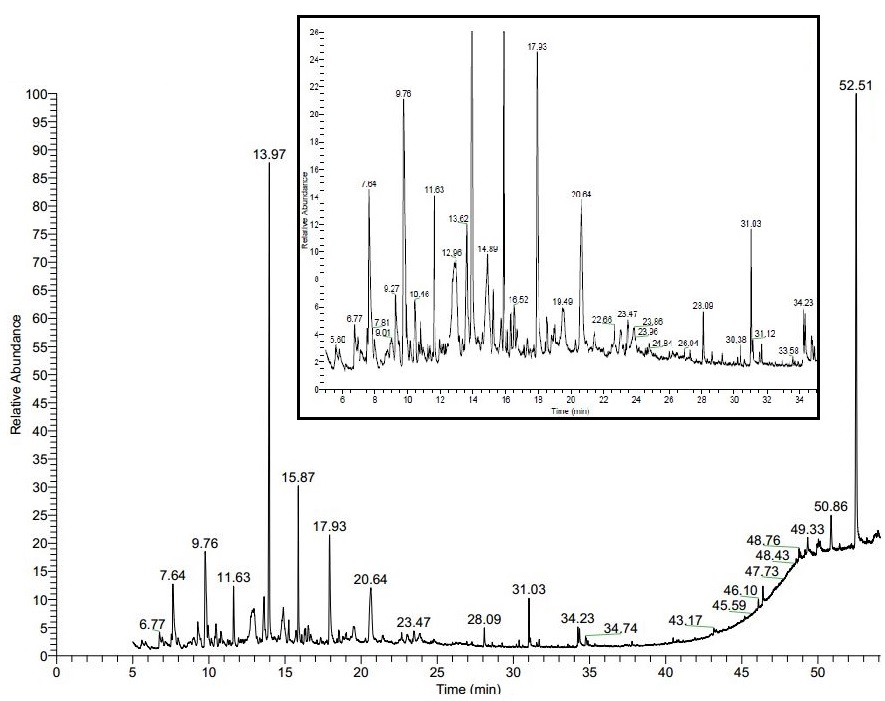

Supplement: Additional file 7: Figure S7. — GCMS spectra of lyophilysed ethanolic flower extract of Lagerstroemia specios (DOCX 125 kb) [file 12906_2016_1495_MOESM7_ESM.docx]
